# Dynamic Modeling and Attitude Decoupling Control for a 3-DOF Flexible Piezoelectric Nano-Positioning Stage Based on ADRC

**DOI:** 10.3390/mi13101591

**Published:** 2022-09-25

**Authors:** Ning Chen, Xianfu Liu

**Affiliations:** 1 College of Mechanical and Electronic Engineering, Shandong University of Science and Technology, Qingdao 266590, China; 2 School of Mechanical Engineering, Shandong University, Jinan 250061, China; 3 School of Mechanical Engineering, Shandong University of Technology, Zibo 255000, China

**Keywords:** piezoelectric, nano-positioning stage, nano-positioning, piezoelectric–mechanical coupling, smart composite structure

## Abstract

The paper proposes a three-degrees-of-freedom flexible nano-positioning stage constructed from compliant flexures and piezoelectric thin-sheet actuators, featuring a compact size and fast dynamic responses, which can be extensively applied to the typical micro/nano-positioning applications. Meanwhile, the dynamic model of the flexible PZT nano-positioning with distributed parameter characteristics is established to distinctly reflect the piezoelectric–mechanical coupling relationship between the four flexible PZT actuators and the three outputs of such a system. Furthermore, the attitude decoupling control for the 3-DOF flexible piezoelectric nano-positioning stage is achieved by the Active Disturbance Rejection Control (ADRC) method to compensate for the positioning errors in the actual positioning process. After this, a real-time experimental apparatus with two Position-Sensitive Detectors (PSDs) is also proposed and fabricated to test the three outputs of the flexible piezoelectric thin-sheet (PZT-5A) nano-positioning stage and validate the effectiveness of the dynamic modeling method and attitude decoupling control in the piezoelectric nano-positioning stage ranges.

## 1. Introduction

Micro-actuators based on piezoelectric materials to efficiently convert electrical energy into ultra/high-precision mechanical displacement have been widely employed in the frontier scientific and technological areas, such as micro/nano-manipulation [1], micro-mirrors [2], nano-machining [3], and biomedical imaging [4]. In particular, many researchers have made great contributions to the design and application of flexure-based micro/nano-motion systems, which directly or indirectly promotes the rapid and successful development of precision engineering applications [5,6]. For example, the piezoelectrically actuated nano-positioning stage was designed and implemented in the hope of obtaining better characteristics [7,8]. Meanwhile, a novel 3-DOF stage with constant-force compliant parallel mechanisms can realize the decoupled constant-force motion requirements in three motion directions [9], as well as the 2-DOF constant-force compliant mechanism gripper [10]. More recently, a compact parallel double parallelogram flexure mechanism with a general beam shape is proposed to offer the desirable performance characteristics, including the independent bearing direction stiffness, robustness against buckling, compact size, and suppression of motion error [11]. Furthermore, the analytical model of the dual-axis compliant micro-manipulator driven by two piezoelectric stack actuators with an asymmetric compliant structure is established by the pseudo-rigid-body model and compliance matrix modeling method [12]. A two-port dynamic stiffness modeling method is developed to describe and capture the kinetostatics and dynamics of many planar compliant mechanisms with very few degrees of freedom [13].

Besides the existing compliant mechanism-based micro/nano-positioning systems investigated above, many alternative methods with compact size, low cost, large stroke, and a high response were also explored, due to the advantages of flexible piezoelectric (thin or thick film) micro-actuators [14]. For example, a two-axis compact scanning stage driven by PZT actuators is proposed, which can be integrated into a laser projection module [15]. Meanwhile, a two-axis optical scanner constructed from a stainless-steel substrate and piezoelectric thin-sheet actuators is proposed and fabricated as an alternative with a large mirror size and scanning angle [16]. After this, an ultrathin XY nano-positioning platform constructed from a piezoelectric thin sheet by an ultrasonic milling process was designed and applied to atomic force microscopy [17,18]. In addition, a $H_{\infty }$ hybrid sensitivity controller is proposed to suppress the tilt of the micro-mirror in both directions of motion, to maintain the pure translational motion, and to eliminate external interference and internal structure deterioration [19]. In spite of a twisting control scheme with a PID sliding surface applied to a torsional micro-mirror to enhance the transient response and high-positioning performance, the chattering problem still exists in closed-loop control systems [20]. Note that the flexible systems present new challenges to the modeling and control on account of the distributed-parameter characteristics and the coupling effect of flexible micro/nano-actuators, which is significantly different from the traditional micro/nano-motion systems driven by PZT stack actuators [21,22,23]. In addition, active disturbance rejection control (ADRC) becomes a popular control method in robot applications, with advantages of simple implementation, strong robustness [24,25], and ability to suppress various uncertainties and disturbances [26,27].

This paper establishes the comprehensive dynamic model of the 3-DOF flexible piezoelectric thin-sheet nano-stage with the transformation to a lumped-parameter system, which can distinctly reflect the piezoelectric–mechanical coupling relationship between the four PZT actuators and the three outputs. Furthermore, the attitude decoupling control for the 3-DOF flexible piezoelectric nano-positioning stage is achieved by the linear Active Disturbance Rejection Control (ADRC) to compensate for the positioning errors caused by the nonlinearity, strong coupling, and all uncertainties of the continuously distributed-parameter device in a real-time experimental apparatus including two Position-Sensitive Detectors (PSDs).

The system is described in Section 2, and the dynamic modeling method of the continuous distributed nano-stage is presented in Section 3. Section 4 introduces the attitude decoupling control for the 3-DOF flexible nano-positioning stage. Finally, the simulations, experiments, and the proposed closed-loop control of the flexible nano-positioning stage inside a laser testing system are illustrated in Section 5.

## 2. Problem Statement

**Figure 1:**
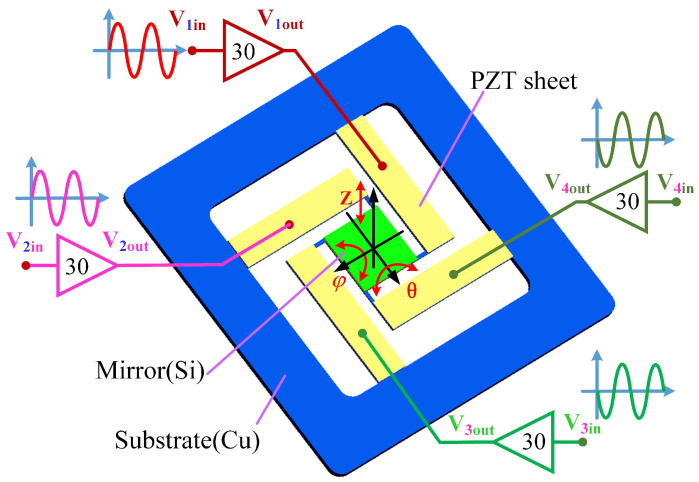
Working principle of the 3-DOF flexible piezoelectric nano-positioning stage.

**Figure 2:**
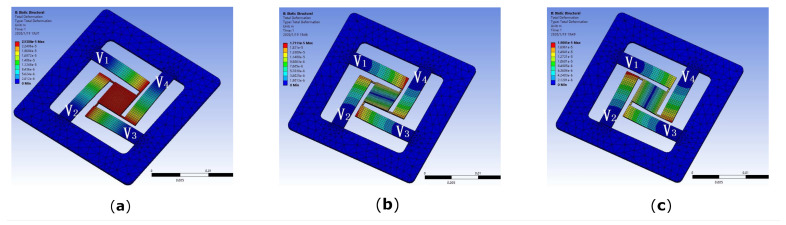
Three- degrees-of-freedom flexible piezoelectric nano-positioning stage. (**a**) Displacement *Z*. (**b**) Rotation angle θ. (**c**) Rotation angle φ.

In this study, four flexible piezoelectric thin-sheet micro-actuators are uniformly employed by the 3-DOF piezoelectric nano-positioning stage to generate a translational displacement (*Z*) and two rotational angles (φ,θ), as depicted in Figure 1, which are composed of piezoelectric thin sheets and copper substrate that are bonded by the adhesive layers. In particular, the piezoelectric nano-positioning stage naturally generates the translational displacement (*Z*), while the four PZT micro-actuators are applied with the same electric fields, given in Figure 2a and Table 1; the proposed system generates the rotational angles (θ, φ), while two adjacent PZT micro-actuators are applied with the same electric fields, and the other two adjacent PZT micro-actuators are applied with opposite electric fields, as shown in Figure 2b,c and Table 1.

The flexible PZT thin-sheet nano-positioning stage featured with the distributed-parameter properties and piezoelectric–mechanical coupling effect is significantly different from the traditional flexure-based micro/nano-motion systems driven by PZT stack actuators and also presents challenges to the MIMO modeling method, due to non-linear, strong coupling, overdrive, and various uncertainties, which can be applied to the model-based control of such systems.

## 3. Dynamic Modeling

**Figure 3:**
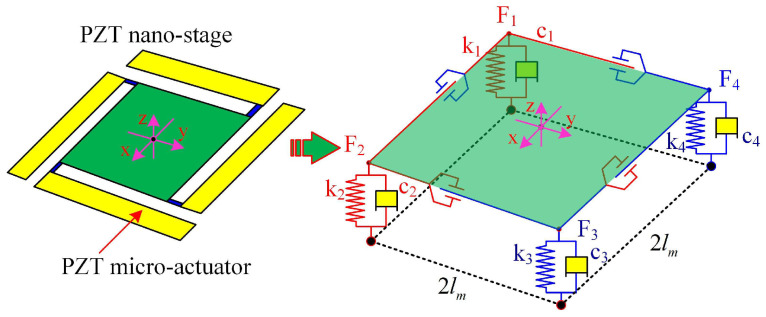
Schematic diagram of the flexible piezoelectric nano-positioning stage.

**Figure 4:**
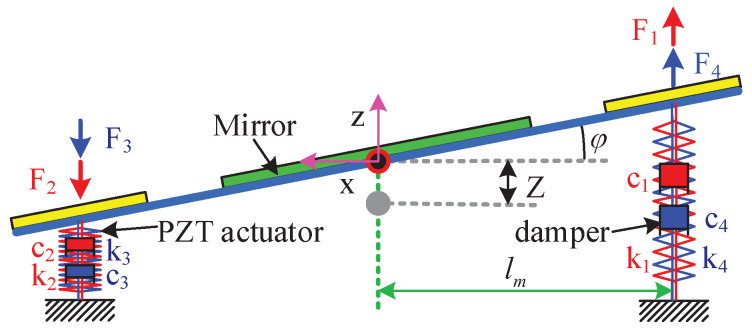
Schematic diagram of the flexible piezoelectric nano-positioning stage.

Based on the conditions of the holonomic mechanical system, we establish a comprehensive dynamic model of the continuous distributed PZT nano-positioning stage driven by flexible PZT thin-sheet micro-actuators by Lagrange’s equations of the second kind, without having to consider the coupling effects between multiple piezoelectric micro-actuators. Meanwhile, the flexible piezoelectric nano-positioning stage can be simplified to a three-dimensional spring–mass–damper system, as in Figure 3. Then, the three motion outputs (*Z*, φ, θ) of the flexible piezoelectric nano-positioning stage are selected as the generalized coordinates $q_{j}$ ($q_{1}=Z$, $q_{2}=\varphi$, $q_{3}=\theta$), and the general formula of the Lagrange equations is

(1) $$\frac{d}{dt}\left(\frac{\partial L}{\partial {\dot{q}}_{j}}\right)-\frac{\partial L}{\partial q_{j}}=Q_{j}\;\left(j=1,2,3\right),$$

where L(=T−V) is the Lagrange function, and $Q_{j}$ are the equivalent generalized forces. *T* and *V* are the equivalent kinetic energy and potential energy of the continuous distributed system, respectively. The distributed-parameter piezoelectric nano-positioning stage is transformed into a lumped-parameter system as depicted in Figure 4, and then the equivalent kinetic energy and potential energy of the piezoelectric nano-positioning stage are obtained as follows:

(2) $$T=\frac{1}{2}m{\dot{Z}}^{2}+\frac{1}{2}J{\dot{\varphi }}^{2}+\frac{1}{2}J{\dot{\theta }}^{2},$$

(3) $$V=\frac{1}{2}k\Delta {d_{1}}^{2}+\frac{1}{2}k\Delta {d_{2}}^{2}+\frac{1}{2}k\Delta {d_{3}}^{2}+\frac{1}{2}k\Delta {d_{4}}^{2},$$

where *k* and *m* are the equivalent stiffness and the equivalent mass of PZT micro-actuators, which can be obtained from Appendix A and [22], respectively. $J(=\frac{ml_{m}^{2}}{3})$ is the equivalent moment of inertia of such a flexible system, and $\Delta d_{i}$ (i=1,2,3,4) are the equivalent deformations of piezoelectric thin-sheet micro-actuators under external forces in [28]. $l_{m}$ is the distance between the PZT micro-actuators and the center of the PZT thin-sheet nano-positioning stage in Figure 4.

Note that the four terminal deformations of the PZT micro-actuators must be kept in a plane, which reflects the piezoelectric–mechanical coupling relationship. Then, we obtain the attitude relationship between the nano-positioning stage outputs and the deformations:

(4) $$\left[\begin{array}{c} Z\\ \varphi \\ \theta \end{array}\right]=\left[\begin{array}{cccc} \frac{1}{4} & \frac{1}{4} & \frac{1}{4} & \frac{1}{4}\;\\ \frac{1}{l_{m}} & -\frac{1}{l_{m}} & -\frac{1}{l_{m}} & \frac{1}{l_{m}}\;\\ -\frac{1}{l_{m}} & -\frac{1}{l_{m}} & \frac{1}{l_{m}} & \frac{1}{l_{m}}\; \end{array}\right]\left[\begin{array}{c} \Delta d_{1}\\ \Delta d_{2}\\ \Delta d_{3}\\ \Delta d_{4} \end{array}\right].$$

Similarly, the deformations ($\Delta d_{i},i=1,2,3,4$) of PZT micro-actuators can also be expressed by the outputs of the flexible PZT nano-positioning stage.

(5) $$\left[\begin{array}{c} \Delta d_{1}\\ \Delta d_{2}\\ \Delta d_{3}\\ \Delta d_{4} \end{array}\right]=\left[\begin{array}{ccc} 1 & l_{m} & -l_{m}\\ 1 & -l_{m} & -l_{m}\\ 1 & -l_{m} & l_{m}\\ 1 & l_{m} & l_{m} \end{array}\right]\left[\begin{array}{c} Z\\ \varphi \\ \theta \end{array}\right].$$

Moreover, the generalized forces $Q_{j}$ of the flexible PZT nano-positioning stage are

(6) $$\left\{\begin{array}{c} Q_{1}\;=F_{1}+F_{2}+F_{3}+F_{4}-4c\dot{Z}\;\\ Q_{2}\;=F_{1}l_{m}-F_{2}l_{m}-F_{3}l_{m}+F_{4}l_{m}-4cl_{m}^{2}\dot{\varphi }\;\\ Q_{3}\;=-F_{1}l_{m}-F_{2}l_{m}+F_{3}l_{m}+F_{4}l_{m}-4cl_{m}^{2}\dot{\theta }\; \end{array}\right.$$

where *c* is the equivalent damping coefficient of the PZT thin-sheet micro-actuators in [29], and $F_{i}\left(i=1,2,3,4\right)$ are the equivalent forces, which can be defined as

(7) $$F_{i}=K_{V}V_{i},i=1,2,3,4,$$

where $V_{i}\left(i=1,2,3,4\right)$ is the voltages applied to the electrodes of the PZT micro-actuators, and $K_{V}$ is the electro-mechanical conversion coefficient of the PZT micro-actuators, which can be obtained by referring to Appendix B. Hence, the comprehensive dynamic model of the 3-DOF flexible PZT thin-sheet nano-positioning stage is established by substituting back with Equations (2), (3), (5) and (6) into Equation (1).

(8) $$\left\{\begin{array}{c} m\ddot{Z}+4c\dot{Z}+4kZ=F_{1}+F_{2}+F_{3}+F_{4}\;\\ J\ddot{\varphi }+cl_{m}^{2}\dot{\varphi }+kl_{m}^{2}\varphi =l_{m}\left(F_{1}-F_{2}-F_{3}+F_{4}\right)\;\\ J\ddot{\theta }+cl_{m}^{2}\dot{\theta }+kl_{m}^{2}\theta =l_{m}\left(-F_{1}-F_{2}+F_{3}+F_{4}\right)\; \end{array}\right.$$

Furthermore, the comprehensive dynamic equations of the 3-DOF flexible piezoelectric nano-positioning stage with voltages ($V_{i},i=1,2,3,4$) as inputs can be expressed as

(9) $$\left\{\begin{array}{c} \ddot{Z}+\frac{4c}{m}\dot{Z}+\frac{4k}{m}Z=\frac{K_{V}}{m}\left(V_{1}+V_{2}+V_{3}+V_{4}\right)\;\\ \ddot{\varphi }+\frac{12c}{m}\dot{\varphi }+\frac{12k}{m}\varphi =\frac{3K_{V}}{ml_{m}}\left(V_{1}-V_{2}-V_{3}+V_{4}\right)\;\\ \ddot{\theta }+\frac{12c}{m}\dot{\theta }+\frac{12k}{m}\theta =\frac{3K_{V}}{ml_{m}}\left(-V_{1}-V_{2}+V_{3}+V_{4}\right)\; \end{array}\right.$$

It can be clearly observed that each output of the flexible piezoelectric nano-positioning stage is controlled by multiple input voltages, which distinctly reflects the piezoelectric–mechanical coupling characteristics of the 3-DOF flexible piezoelectric nano-positioning stage, where the model can be used for the model-based attitude decoupling control of such MIMO systems.

## 4. Attitude Decoupling Control of the Piezoelectric Nano-Positioning Stage

**Figure 5:**
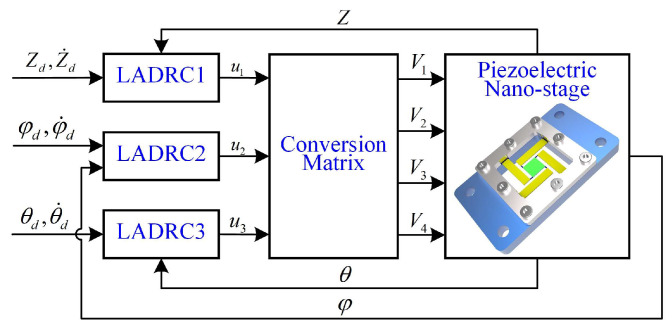
Block diagram of the attitude decoupling control system.

The designed 3-DOF flexible piezoelectric thin-sheet nano-positioning stage above is considered as an over-constrained MIMO system in the scope of control and manipulation. It is noted that non-linearities, uncertainties, strong axis couplings, and various external disturbances exist in the nano-positioning stage in different work conditions, which poses severe challenges for multi-DOF robust control design for such systems. To achieve this goal, an ADRC-based MIMO attitude decoupling control is designed in this work, which is depicted in Figure 5, to provide three virtual control inputs ($u_{i},i=1,2,3$) to control the multi-DOF nano-positioning system.

### 4.1. Conversion Matrix of Control Variables

Referring to Equations (8) and (9), we obtain the relationships between the virtual control input $u_{i}\left(i=1,2,3\right)$ and the equivalent force $F_{i}\left(i=1,2,3,4\right)$, as well as input voltages $V_{i}\left(i=1,2,3,4\right)$.

(10) $$\begin{array}{ccc} \left[\begin{array}{c} \frac{1}{m}u_{1}\\ \frac{l_{m}}{J}u_{2}\\ \frac{l_{m}}{J}u_{3} \end{array}\right] & = & \left[\begin{array}{cccc} \frac{1}{m} & \frac{1}{m} & \frac{1}{m} & \frac{1}{m}\;\\ \frac{l_{m}}{J} & -\frac{l_{m}}{J} & -\frac{l_{m}}{J} & \frac{l_{m}}{J}\;\\ -\frac{l_{m}}{J} & -\frac{l_{m}}{J} & \frac{l_{m}}{J} & \frac{l_{m}}{J}\; \end{array}\right]\left[\begin{array}{c} F_{1}\\ F_{2}\\ F_{3}\\ F_{4} \end{array}\right]\\ & = & \left[\begin{array}{cccc} \frac{K_{V}}{m} & \frac{K_{V}}{m} & \frac{K_{V}}{m} & \frac{K_{V}}{m}\;\\ \frac{K_{V}l_{m}}{J} & -\frac{K_{V}l_{m}}{J} & -\frac{K_{V}l_{m}}{J} & \frac{K_{V}l_{m}}{J}\;\\ -\frac{K_{V}l_{m}}{J} & -\frac{K_{V}l_{m}}{J} & \frac{K_{V}l_{m}}{J} & \frac{K_{V}l_{m}}{J}\; \end{array}\right]\left[\begin{array}{c} V_{1}\\ V_{2}\\ V_{3}\\ V_{4} \end{array}\right]. \end{array}$$

Moreover, the relationship between the equivalent deformations ($\Delta d_{i},i=1,2,3,4$) of the piezoelectric thin-sheet micro-actuators and input voltages $\left(V_{i},i=1,2,3,4\right)$ can be expressed as

(11) $$\Delta d_{i}=K_{d}V_{i},i=1,2,3,4,$$

where $K_{d}$ is the electro-deformation conversion coefficient of the PZT micro-actuators. Substituting back with Equation (11) into Equation (5) leads to the relationship between the input voltages and the outputs:

(12) $$\left[\begin{array}{c} V_{1}\\ V_{2}\\ V_{3}\\ V_{4} \end{array}\right]=\left[\begin{array}{ccc} \frac{1}{K_{d}} & \frac{l_{m}}{K_{d}} & -\frac{l_{m}}{K_{d}}\;\\ \frac{1}{K_{d}} & -\frac{l_{m}}{K_{d}} & -\frac{l_{m}}{K_{d}}\;\\ \frac{1}{K_{d}} & -\frac{l_{m}}{K_{d}} & \frac{l_{m}}{K_{d}}\;\\ \frac{1}{K_{d}} & \frac{l_{m}}{K_{d}} & \frac{l_{m}}{K_{d}}\; \end{array}\right]\left[\begin{array}{c} Z\\ \varphi \\ \theta \end{array}\right].$$

With Equations (5) and (10)–(12), we eventually obtain the conversion matrix between input control voltages ($V_{i},i=1,2,3,4$) and virtual control variables ($u_{i},i=1,2,3$).

(13) $$\left[\begin{array}{c} V_{1}\\ V_{2}\\ V_{3}\\ V_{4} \end{array}\right]=\left[\begin{array}{ccc} \frac{m}{4K_{V}} & \frac{ml_{m}}{12K_{V}} & -\frac{ml_{m}}{12K_{V}}\;\\ \frac{m}{4K_{V}} & -\frac{ml_{m}}{12K_{V}} & -\frac{ml_{m}}{12K_{V}}\;\\ \frac{m}{4K_{V}} & -\frac{ml_{m}}{12K_{V}} & \frac{ml_{m}}{12K_{V}}\;\\ \frac{m}{4K_{V}} & \frac{ml_{m}}{12K_{V}} & \frac{ml_{m}}{12K_{V}}\; \end{array}\right]\left[\begin{array}{c} \frac{1}{m}u_{1}\;\\ \frac{l_{m}}{J}u_{2}\;\\ \frac{l_{m}}{J}u_{3}\; \end{array}\right].$$

### 4.2. Linear Active Disturbance Rejection Control

Based on the comprehensive dynamic model (13), the ADRC method is implemented in the over-driven, MIMO high-precision control diagram to achieve the attitude decoupling control of the 3-DOF flexible piezoelectric nano-positioning stage, due to its strong disturbance rejection capabilities and prospective control performance. In particular, the ADRC idea is to design an extended state observer, which can estimate the total disturbance of the system, which is compensated for in the closed-loop control.

In this work, couplings between different DOFs are considered as disturbances such that decoupling can be realized by the ADRC of each axis. To design the linear ADRC for the MIMO system, we first take the output (*Z*) dynamic model as an example to illustrate the attitude decoupling control of the flexible piezoelectric nano-positioning stage with the LADRC. For the convenience of description, the equation parameter *Z* can be replaced by *y*, and the first dynamic equation of the Equation (8) is rewritten as

(14) $$\ddot{y}=f\left(y,\dot{y},\omega \right)+b_{0}u,$$

where $f\left(y,\dot{y},\omega \right)$ is the total disturbance, including the internal dynamics $\frac{4c}{m}\dot{y}+\frac{4k}{m}y$ and the external disturbance ω(t); $b_{0}=\frac{1}{m}$. We assume that the state variables as $x\left(t\right)={\left[x_{1},x_{2},x_{3}\right]}^{T}={\left[y,\dot{y},f\right]}^{T}$. Hence, the dynamic model of the PZT thin-sheet nano-positioning stage can be rewritten as the following extended system:

(15) $$\left\{\begin{array}{c} \dot{x}=Ax+Bu+E\dot{f}\\ y=Cx \end{array}\right.$$

where

$$A=\left[\begin{array}{ccc} 0 & 1 & 0\\ 0 & 0 & 1\\ 0 & 0 & 0 \end{array}\right],\;B=\left[\begin{array}{c} 0\\ b_{0}\\ 0 \end{array}\right],\;E=\left[\begin{array}{c} 0\\ 0\\ 1 \end{array}\right],\;C={\left[\begin{array}{c} 1\\ 0\\ 0 \end{array}\right]}^{T}.$$

#### 4.2.1. Linear Extended State Observer Design

The key of ESO is to estimate the total disturbance of the flexible piezoelectric nano-positioning stage, including the dynamic model parameter perturbations, structural uncertainties, and external disturbances. Then, the extended state observer is

(16) $$\left\{\begin{array}{c} \dot{\hat{z}}=A\hat{z}+Bu+L\dot{f}\\ y=C\hat{z} \end{array}\right.$$

where $z={\left[z_{1},z_{2},z_{3}\right]}^{T}$ is the estimated state for $x\left(t\right)={\left[y,\dot{y},f\right]}^{T}$, and $L={\left[l_{1},l_{2},l_{3}\right]}^{T}$ is the observer gain. Referring to the equivalent bandwidth scaling method [30], the observer gain can be parameterized as:

(17) $$L={\left[l_{1},l_{2},l_{3}\right]}^{T}={\left[3\omega_{o},3\omega_{o}^{2},\omega_{o}^{3}\right]}^{T},$$

where $\omega_{o}$ is defined as the observer bandwidth, which can be appropriately selected to ensure the characteristic polynomial $s^{3}+l_{1}s^{2}+l_{2}s+l_{3}$ Hurwitz.

#### 4.2.2. Feedback Controller Design

**Figure 6:**
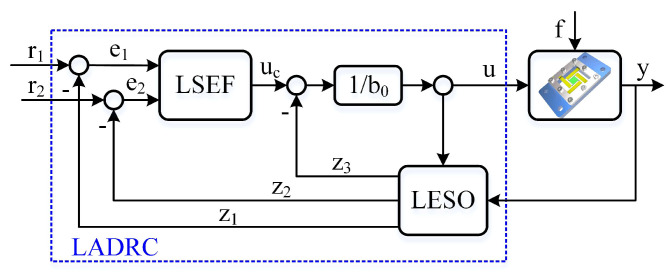
Block diagram of the linear ADRC.

With the estimation of the extended state observer (16), we design a PD feedback control law, also noted as linear state extended feedback (LSEF in Figure 6), to compensate for the estimated total disturbance and control the whole system.

(18) $$u=\frac{u_{c}-z_{3}}{b_{0}},$$

with

(19) $$u_{c}=k_{1}\left(r_{1}-z_{1}\right)+k_{2}\left(r_{2}-z_{2}\right),$$

where $r_{1}$ and $r_{2}$ are the reference signals of the displacement and velocity in the z-axis, respectively; $k_{1}$ and $k_{2}$ are the state feedback controller gains. By choosing

$$k_{1}=\omega_{c}^{2},k_{2}=2\omega_{c},$$

the transfer function of the PZT nano-positioning stage in the z-axis can be transformed into

(20) $$G_{c}=\frac{k_{1}}{s^{2}+k_{2}s+k_{1}}=\frac{\omega_{c}^{2}}{{\left(s+\omega_{c}\right)}^{2}},$$

where $\omega_{c}$ is considered as the closed-loop feedback controller bandwidth. Then, we can select an appropriate $\omega_{c}$ to obtain the desired control performance.

In practice, the observer bandwidth $\omega_{o}$ is selected as 3 to 5 times the value of $\omega_{c}$, where the attitude decoupling control of flexible PZT nano-positioning stages can be achieved with requirements.

## 5. Detection Method and Results

**Figure 7:**
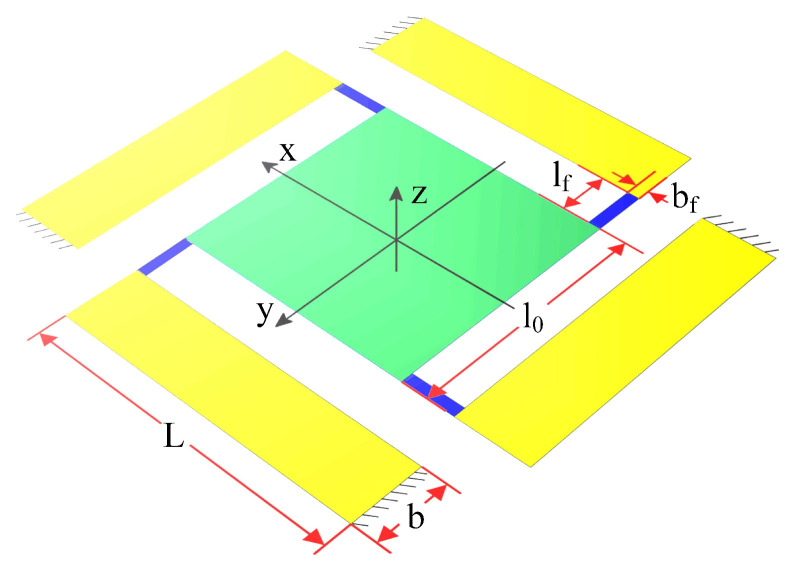
Structural parameters of the piezoelectric nano-positioning stage.

We design and fabricate a sample of the flexible piezoelectric nano-positioning stage based on the structural parameters in Table 2 and Figure 7, which is composed of PZT thin sheets (PZT-5A, Table 3) and a copper substrate, to further verify the effectiveness of the established model and attitude decoupling control method. Specifically, the piezoelectric thin sheets are bonded by silver glue at room temperature, which can restrain the significant residual stress, compared with traditional manufacturing and assembly methods. Meanwhile, we propose a novel detection method including two low-cost Position-Sensitive Detectors (PSDs) to detect the three outputs of the flexible piezoelectric nano-positioning stage in real time, which overcomes the disadvantages of the high cost, large size, and high installation accuracy of traditional detection methods such as the laser interferometer and grating ruler. In addition, the photosensitive area of PSD100-SPB is 10 mm × 10 mm with 5 μm position resolution and the wavelength of the laser is 650 nm.

### 5.1. Detection Method and Experimental Setup

**Figure 8:**
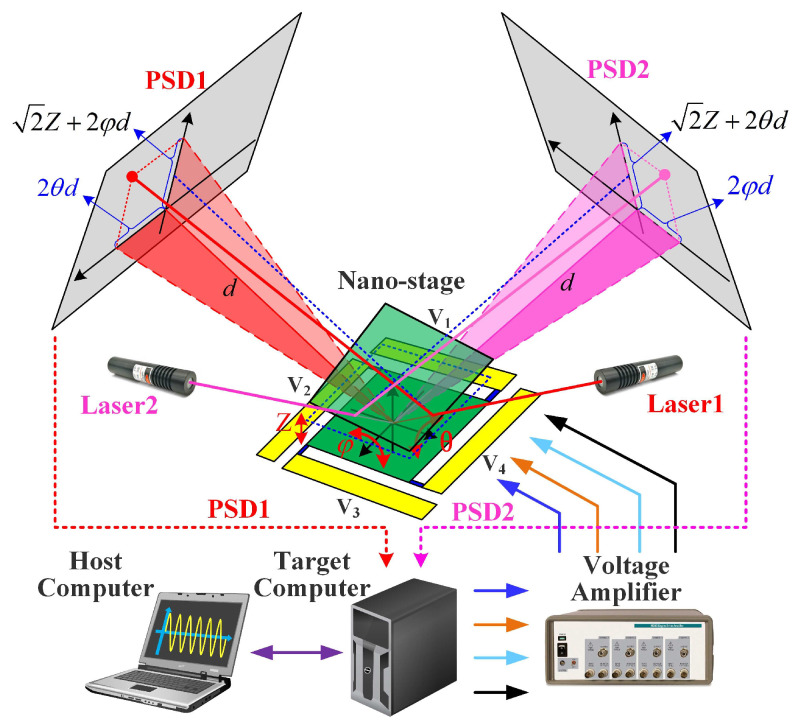
Schematic diagram of the detection method and the experimental setup.

**Figure 9:**
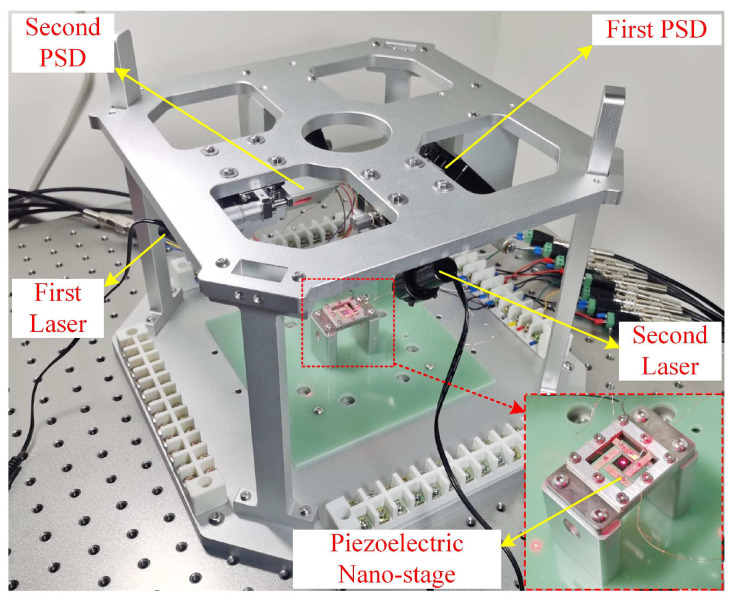
Apparatus of experiments.

The schematic diagram of the novel detection method based on double Position-Sensitive Detectors (PSDs) is depicted in Figure 8. Furthermore, the experimental instrument is assembled as shown in Figure 9.

### 5.2. Experimental Results

**Figure 10:**
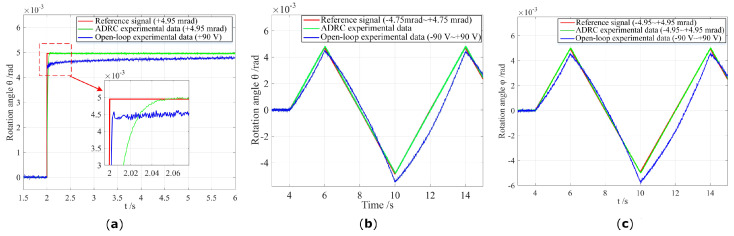
Attitude decoupling control for the 3-DOF flexible piezoelectric nano-positioning stage. (**a**) Step response θ. (**b**) Rotation angle θ. (**c**) Rotation angle φ.

As depicted in Figure 10, real-time experiments under different conditions and signals are conducted to verify the effectiveness of the comprehensive dynamic model and attitude decoupling control method. For example, the actual output values (θ,φ in the range of ±4.9mrad, *Z* in the range of ±23.5 μm) of the flexible PZT nano-positioning stage in the voltage range from −90 V to +90 V are basically consistent with the theoretical and reference values. Moreover, the attitude decoupling control method using LADRCs simultaneously allows the proposed system the obtain high-speed and high-position accuracy, where it effectively eliminates and compensates for the errors caused by the hysteresis and creep of piezoelectric thin-sheet micro-actuators. The simulation results of the piezoelectric nano-positioning stage have been shown in [22], where the uniaxial tracking error is approximately 0.86% in tracking a sinusoidal signal (50 Hz) with this ADRC method.

**Figure 11:**
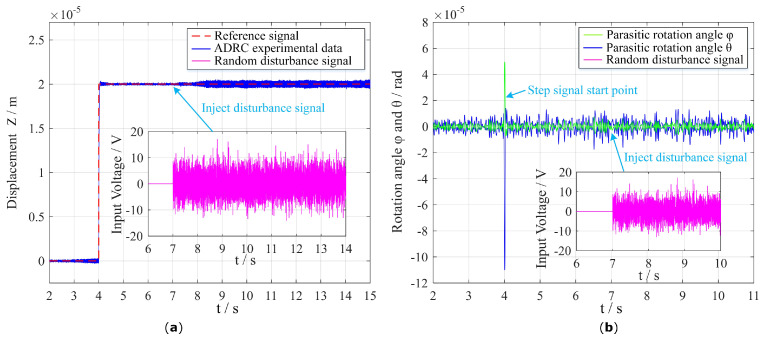
Three output closed-loop testing signals of PZT nano-positioning stages under step and disturbance signals. (**a**) Displacement *Z*. (**b**) Parasitic rotation angle φ and θ.

Due to the limitation of the output power of the voltage amplifier, we can only test the suppression effect under a 10 V disturbance signal. It is worth noting that we simultaneously obtain the three output closed-loop detection signals of the flexible PZT nano-positioning stage with the step and disturbance signal applied to the electrodes of PZT thin sheets, as shown in Figure 11, which further demonstrate and verify the high-speed, high-precision, and anti-interference characteristics of the attitude decoupling control method using LADRCs. In addition, the difference in the fluctuation range between the parasitic rotation angle and parasitic rotation angle was mainly caused by the inconsistent preparation of the four PZT micro-actuators, where piezoelectric sheets are bonded with conductive silver glue.

**Figure 12:**
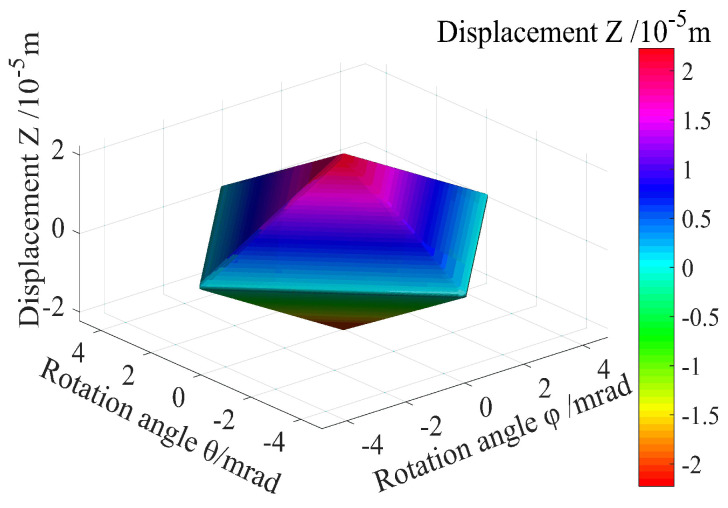
Three motion ranges of the flexible piezoelectric nano-positioning stage under −90V∼+90V.

It is also observed that the three total ranges of flexible piezoelectric nano-positioning stages are mutually limited, as depicted in Figure 12, where the stroke of each piezoelectric thin-sheet micro-actuator used to drive the proposed systems is limited due to safety considerations of piezoelectric thin sheets. We compared the proposed design and some existing works in Table 4.

## 6. Conclusions

This paper established the comprehensive dynamic model of a 3-DOF nano-positioning stage driven by four uniformly distributed piezoelectric thin-sheet micro-actuators, which helps to study and reflect the piezoelectric–mechanical coupling characteristics of the flexible piezoelectric nano-positioning stage accurately. In particular, the distributed-parameter piezoelectric nano-positioning stage is transformed into a lumped-parameter system by solving the equivalent parameters of distributed piezoelectric micro-actuators, further providing theoretical support for the model-based control method of such systems. The effectiveness of the established model and the attitude decoupling control model was comprehensively demonstrated by experiments and simulations. In addition, the 3-DOF flexible piezoelectric thin-sheet nano-positioning stage, featuring a compact size, low cost, and high dynamic responses, provides an alternative to flexure-based micro/nano-motion stages at a low-frequency range, and the ultra-precision control method of flexible nano-positioning stages will be further explored in future works.

## Figures and Tables

**Table 1:** Relationship between output of nano-positioning stage and voltage of piezoelectric actuators.

Output	$V_{1}$	$V_{2}$	$V_{3}$	$V_{4}$
Z	+(−)V	+(−)V	+(−)V	+(−)V
θ	+(−)V	+(−)V	−(+)V	−(+)V
φ	+(−)V	−(+)V	−(+)V	+(−)V

**Table 2:** Structural parameters of the piezoelectric nano-positioning stage.

Dimensional Parameters	Value (mm)
Length of the micro-actuator (L)	11.3
Width of the micro-actuator (b)	2.5
Thickness of the substrate layer ($h_{s}$)	0.1
Thickness of the PZT-5A ($h_{p}$)	0.2
Length of the flexure hinge ($l_{f}$)	1.0
Width of the flexure hinge ($b_{f}$)	0.3
Thickness of the flexure hinge ($t_{f}$)	0.1
Length of the center mirror ($l_{0}$)	5.0
Thickness of the center mirror ($h_{si}$)	0.5

**Table 3:** Parameters of PZT-5A.

Mechanical Properties	PZT-5A
Compliance of the PZT $\left(10^{-12}m^{2}/N\right)$	Value
$s_{11}^{p}$	16.40
$s_{12}^{p}$	−5.40
$s_{13}^{p}$	−7.22
$s_{33}^{p}$	18.80
$s_{44}^{p}$	47.50
$s_{66}^{p}$	44.30
Piezoelectric strain coefficient $\left(10^{-12}m^{2}/V\right)$	Value
$d_{15}$	584
$d_{31}$	−171
$d_{33}$	374

**Table 4:** Comparison of the proposed design and some existing works.

Sample Parameters	Proposed Design	Ref. [19]	Ref. [20]	NS−RB4−014	S−340
Structure size (mm)	17.6×17.6×0.6	52×52×0.5	59×59×1	ϕ52×60	ϕ75×90
Static gain	±90 *V*	−200 V∼+500 *V*	±200 *V*	−30 V∼+150 *V*	−20 V∼+120 *V*
Z (μm)	49.8	−−	26.5	−−	−−
Y (μm)	−−	6	6.22	−−	−−
X (μm)	−−	6	5.27	−−	−−
θ (mrad)	10.2	−−	0.60	4	2
φ (mrad)	10.3	−−	0.884	4	2
Frequency (Hz)					
z-Axis	482	530.9	845	−−	−−
x/y-Axis	−−	1163	−−	−−	−−
θ/x-Axis	(721)	−−	1850	900	1400
φ/y-Axis	(788)	−−	1850	900	1400

**Figure A1:**
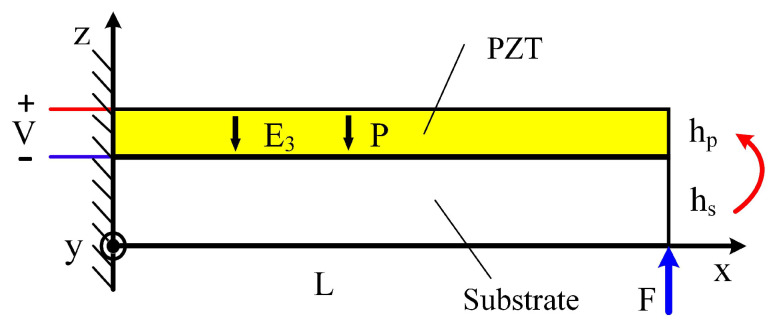
Deflection of the piezoelectric beam.

## Abbreviations

The following abbreviations are used in this manuscript:

DOF	Degree of Freedom
ADRC	Active Disturbance Rejection Control
PSD	Position-Sensitive Detector
ESO	Extended State Observer

## Nomenclature

$\varepsilon_{E}$	strain slope of unimorph benders
*b*	width of bender
*d*	center distance between detector (PSD) and mirror
$d_{31}$	coefficient of piezoelectric strains
$E_{3}$	electric field of unimorph benders
*F*	force (general)
$h_{i}$	thickness of each layer of piezoelectric benders (defined by index)
*L*	length of bender
*P*	polarization direction
$s_{ij}^{p},s_{ij}^{s}$	compliance coefficient of piezo and substrate material
$V_{i}$	voltage applied to unimorph benders (defined by index)
x,y,z	space coordinates

## Appendix A. Electro-Mechanical Conversion Coefficient

The deflection or deformation of PZT thin-sheet micro-actuators under electric fields can be found in [28].

(A1) $$\begin{array}{ccc} w_{E}\left(L\right) & = & {\left.\frac{1}{2}\varepsilon_{E}x^{2}\right|}_{x=L}=\frac{1}{2}\varepsilon_{E}L^{2}. \end{array}$$

Then, the equivalent stiffness (*k*) of flexible PZT thin-sheet micro-actuators with distributed-parameter characteristics is

(A2) $$k=\frac{F}{w\left(L\right)}=\frac{\frac{3\varepsilon_{E}I_{y}}{2s_{11}^{s}L}}{\frac{1}{2}\varepsilon_{E}L^{2}}=\frac{3I_{y}}{s_{11}^{s}L^{3}}.$$

## Appendix B. Equivalent Stiffness

As depicted in Figure A1, the equivalent force *F* of the flexible piezoelectric thin-sheet micro-actuators of the piezoelectric nano-positioning stage under electric fields can be obtained obtained in [22], and we define the equivalent force (*F*) as follows:

(A3) $$F=\frac{3\varepsilon_{E}I_{y}}{2s_{11}^{s}L}=K_{V}V.$$

(A4) $$\begin{array}{ccc} I_{y} & = & \int_{\beta_{E}}^{h_{s}+\beta_{E}}bz^{2}dz+\frac{s_{11}^{s}}{s_{11}^{p}}\int_{h_{s}+\beta_{E}}^{h_{p}+h_{s}+\beta_{E}}bz^{2}dz\\ & = & \frac{bK\left(\begin{array}{c} E_{s}^{2}Q{\left(1-v_{p}\right)}^{2}+E_{p}^{2}Y{\left(1-v_{s}\right)}^{2}-2E_{p}E_{s}R\left(1-v_{p}\right)\left(1-v_{s}\right) \end{array}\right)}{36{\left(h_{s}+h_{p}\right)}^{2}s{{}_{11}^{s}}^{2}s{{}_{11}^{p}}^{3}{\left(\begin{array}{c} E_{s}h_{s}\left(1-v_{p}\right)+E_{p}h_{p}\left(1-v_{s}\right) \end{array}\right)}^{2}}, \end{array}$$

where

$$\begin{array}{ccc} K & = & h_{p}^{4}s{{}_{11}^{s}}^{2}+\left(4h_{p}^{3}h_{s}+6h_{p}^{2}h_{s}^{2}+4h_{p}h_{s}^{3}\right)s_{11}^{s}s_{11}^{p}+h_{s}^{4}s{{}_{11}^{p}}^{2},\\ Q & = & \left(h_{p}^{3}s_{11}^{s}+h_{s}\left(3h_{p}^{2}+6h_{p}h_{s}+4h_{s}^{2}\right)s_{11}^{p}\right)s{{}_{11}^{s}}^{2},\\ Y & = & \left(h_{s}^{3}s_{11}^{p}+h_{p}\left(3h_{s}^{2}+6h_{s}h_{p}+4h_{p}^{2}\right)s_{11}^{s}\right)s{{}_{11}^{p}}^{2},\\ R & = & \left(h_{p}^{3}s_{11}^{s}+h_{s}^{3}s_{11}^{p}\right)s_{11}^{s}s_{11}^{p}. \end{array}$$

Hence, the flexible PZT nano-positioning stage can be transformed into a lumped-parameter system, and we obtain the electro-mechanical conversion coefficient ($K_{V}$), which helps to achieve the model-based control of such flexible systems.

(A5) $$K_{V}=-\frac{9d_{31}h_{s}\left(h_{s}+h_{p}\right)s_{11}^{p}I_{y}}{L\left(h_{p}^{4}s{{}_{11}^{s}}^{2}+2h_{s}h_{p}\left(\begin{array}{c} 2h_{s}^{2}+3h_{s}h_{p}+2h_{p}^{2} \end{array}\right)s_{11}^{s}s_{11}^{p}+h_{s}^{4}s{{}_{11}^{p}}^{2}\right)}.$$
